# Off-label use of combined antiretroviral therapy, analysis of data collected by the Italian Register for HIV-1 infection in paediatrics in a large cohort of children

**DOI:** 10.1186/s12879-022-07026-w

**Published:** 2022-01-15

**Authors:** Elena Chiappini, Catiuscia Lisi, Vania Giacomet, Paola Erba, Stefania Bernardi, Paola Zangari, Antonio Di Biagio, Lucia Taramasso, Carlo Giaquinto, Osvalda Rampon, Clara Gabiano, Silvia Garazzino, Claudia Tagliabue, Susanna Esposito, Eugenia Bruzzese, Raffaele Badolato, Domenico Zanaboni, Monica Cellini, Maurizio Dedoni, Antonio Mazza, Andrea Pession, Anna Maria Giannini, Filippo Salvini, Icilio Dodi, Ines Carloni, Salvatore Cazzato, Pier Angelo Tovo, Maurizio de Martino, Luisa Galli, for the Italian Register for HIV Infection in Children, Sara Parigi, Francesca Orlandi, Alessandra de Martino, Raffaella Pinzani, Luisa Abbagnato, Maurizio Ruggeri, Francesco Baldi, Giacomo Faldella, Piergiorgio Chiriacò, Carlo Dessì, Maria Grazia Pantò, Elisa Anastasio, Maria Rita Govoni, Maurizio Bigi, Elisabetta Bondi, Riccardo Borea, Giovanni Cenderello, Donato Tommasi, Ernesto Renato Dalle Nogare, Marcello Saitta, Leonardo Felici, Rita Consolini, Angelo Antonellini, Gianfranco Anzidei, Orazio Genovese, Salvatore Catania, Fabio Natale, Paolina Olmeo, Letizia Cristiano, Vincenzo Portelli, Marco Rabusin, Giada Maria Di Pietro, Leone Fabrizio

**Affiliations:** 1 Paediatric Infectious Diseases Unit, Department of Health Sciences, Anna Meyer Children’s University Hospital, University of Florence, Viale Pieraccini, 24, 50100 Florence, Italy; 2 Paediatric Infectious Diseases Unit, Department of Paediatrics, ASST FBF SACCO, University of Milan, Milan, Italy; 3 Academic Department of Pediatrics (DPUO), Research Unit of Clinical Immunology and Vaccinology, Bambino Gesù Children’s Hospital, IRCCS, Rome, Italy; 4 Infectious Disease Unit, Ospedale Policlinico San Martino, University of Genova, Genova, Italy; 5 Department of Women and Child Health, University of Padova, Padova, Italy; 6 Paediatric Infectious Diseases Unit, Regina Margherita Children’s Hospital, University of Turin, Turin, Italy; 7 Foundation IRCCS Ca’ Granda Ospedale Maggiore Policlinico, Milan, Italy; 8 Paediatric Clinic, Pietro Barilla Children’s Hospital, University of Parma, Parma, Italy; 9 Department of Translational Medical Sciences, Section of Paediatrics, University of Naples Federico II, Naples, Italy; 10 Department of Clinical and Experimental Sciences, University of Brescia, Brescia, Italy; 11 Department On Internal Medicine and Therapeutics, IRCCS Policlinico “S. Matteo” Foundation, University of Pavia, Pavia, Italy; 12 Department of Medical and Surgical Sciences, Section of Paediatric Hemato-Oncology, University of Modena and Reggio Emilia, Modena, Italy; 13 Department of Paediatrics, Ospedale Microcitemico, Cagliari, Italy; 14 Paediatric Unit, “S. Chiara” Hospital, Trento, Italy; 15 Andrea Pession, Paediatric Unit, IRCCS Azienda Ospedaliero-Universitaria, Bologna, Italy; 16 Paediatric Infectious Diseases Unit, University Hospital Policlinico Giovanni XXIII, Bari, Italy; 17 Department of Paediatrics, University of Milan, Niguarda Hospital, Milan, Italy; 18 Department of Mother and Child Health, Salesi Children’s Hospital, Ancona, Italy; 19 Paediatric Unit, Sant’Anna Hospital, Como, Italy

**Keywords:** HIV-1 infection, Children, Antiretroviral therapy, HAART, Off-label therapy

## Abstract

**Background:**

Early start of highly active antiretroviral therapy (HAART) in perinatally HIV-1 infected children is the optimal strategy to prevent immunological and clinical deterioration. To date, according to EMA, only 35% of antiretroviral drugs are licenced in children < 2 years of age and 60% in those aged 2–12 years, due to the lack of adequate paediatric clinical studies on pharmacokinetics, pharmacodynamics and drug safety in children.

**Methods:**

An observational retrospective study investigating the rate and the outcomes of off-label prescription of HAART was conducted on 225 perinatally HIV-1 infected children enrolled in the Italian Register for HIV Infection in Children and followed-up from 2001 to 2018.

**Results:**

22.2% (50/225) of included children were receiving an off-label HAART regimen at last check. Only 26% (13/50) of off-label children had an undetectable viral load (VL) before the commencing of the regimen and the 52.0% (26/50) had a CD4 + T lymphocyte percentage > 25%. At last check, during the off label regimen, the 80% (40/50) of patients had an undetectable VL, and 90% (45/50) of them displayed CD4 + T lymphocyte percentage > 25%. The most widely used off-label drugs were: dolutegravir/abacavir/lamivudine (16%; 8/50), emtricitbine/tenofovir disoproxil (22%; 11/50), lopinavir/ritonavir (20%; 10/50) and elvitegravir/cobicistat/emtricitabine/ tenofovir alafenamide (10%; 10/50). At logistic regression analysis, detectable VL before starting the current HAART regimen was a risk factor for receiving an off-label therapy (OR: 2.41; 95% CI 1.13–5.19; p = 0.024). Moreover, children < 2 years of age were at increased risk for receiving off-label HAART with respect to older children (OR: 3.24; 95% CI 1063–7.3; p = 0.001). Even if our safety data regarding off-label regimens where poor, no adverse event was reported.

**Conclusion:**

The prescription of an off-label HAART regimen in perinatally HIV-1 infected children was common, in particular in children with detectable VL despite previous HAART and in younger children, especially those receiving their first regimen. Our data suggest similar proportions of virological and immunological successes at last check among children receiving off-label or on-label HAART. Larger studies are needed to better clarify efficacy and safety of off-label HAART regimens in children, in order to allow the enlargement of on-label prescription in children.

**Supplementary Information:**

The online version contains supplementary material available at 10.1186/s12879-022-07026-w.

## Introduction

Over the years, a marked evolution of antiretroviral therapy has occurred; starting from 1987 with the introduction of the first antiretroviral (ARV) drug, zidovudine, to 1997 with the introduction of protease inhibitors, up to the most recent ARV classes including fusion inhibitors, integrase inhibitors and CCR5 inhibitors [1]. The progression of combination antiretroviral therapy (cART) has made possible for HIV-1 infection to become a chronic disease. On the other hand, the therapy has become more difficult to manage, especially in children. In these patients, potential issues concern the management of the cART, including poor adherence (especially in adolescents), the high burden of daily pills, the few age-appropriate drug formulations and the differences in pharmacokinetics between children and adults that contribute in determining the effectiveness and safety of the cART itself [2]. According to recent data, nowadays about 12% of cART treated children still experience treatment failure, mainly due to drug resistance or poor adherence to therapy, related to lack of paediatric formulations or poor palatability of liquid formulations [2]. Childhood is also associated with metabolic and pharmacokinetic changes, such as differences in absorption, distribution and elimination of drugs, reduced intestinal motility or slowed gastric emptying [3]. In adolescents, drug interactions (such as with oral contraceptives) and puberty, in addition to weight and age, could influence the dosage of drugs [4].

To date, only 34.4% of ARVs administered in adults are approved for use in children under 2 years of age, and 62% are approved in those under 12 years of age in Europe [5]. The approval for paediatric use of ARVs may be adventurous with variable latency of time compared to the adult. As an example, tenofovir disoproxil fumarate has been approved in 2002 for adults and only in 2012 for children [5]. This has inevitably led to extensive off-label use of drugs in children with perinatal HIV-1 infection by the clinicians, who, moreover, have to deal with few available literature data regarding the off-label use to cART therapies.

In a Spanish multicentric study including 318 perinatally HIV-1-infected children and adolescents off-label use of cART involved the 69% of children [6].

In the present study, data collected from the *Italian Register of HIV infection in Paediatrics* were retrospectively collected and analysed with the aim to evaluate off label use of cART in perinatally HIV-1 infected children in recent years.

## Methods

### Data collection

The *Italian Register for HIV Infection in Paediatrics* is a database concerning cases of children with HIV-1 infection, established in 1985 on the initiative of the Immunology Study Group, within the Italian Society of Paediatrics. Data have been recorded since June 1, 1985. Data of children born before this period, about 13% of the data, have been collected retrospectively [7].

The source of the Register is represented by a network of 106 paediatric clinical centres distributed throughout Italy. These centres, which participate voluntarily, register all children with HIV-1 infection. Two coordination centres are located at the Paediatric Clinic of Infectious Diseases of the Department of Paediatrics of the University of Florence and the Department of Paediatric and Adolescent Sciences of the University of Turin. The Registry includes all children with HIV-1 infection, either born to a mother with HIV-1, or identified at birth or reported later. The data are representative of the entire population of children born to mothers with HIV-1 infection in Italy [7].

Data on mother–child couples are collected using specific reporting and follow-up forms and are processed anonymously, in accordance with the relevant guidelines and regulations.

In particular, basic information regarding the mother include: the mode of infection such as sexual, drug addiction, transfusions, endemic area origin; number of pregnancies; mode of delivery; gestational age and clinical condition of the mother at the time of delivery reported according to the CDC classification system. Since 1989, data concerning the mother include a classification of women treated and untreated, since 1993 there is information on the type of therapy, gestational age at the beginning and at the end of the therapy and since 1994, with the introduction of PACTG 076 protocol, it is also indicated whether the treatment has been administered intrapartum or to the newborn. If the treatment has been administered during pregnancy, all information concerning the combination of drugs used is reported in a special section. Information on possible resistance to treatment, concomitant maternal infections and the conformity of therapy is not collected. The immunological condition of the mother, indicated by the CD4^+^ T lymphocyte count, and the viral load (VL) at the time of delivery have been reported since 1996 and are only known in a minority of cases. The information concerning the child reported in the Register, are the following: birth weight, age at first observation, type and duration of breastfeeding and state of infection. Whereas, in the follow-up records there are information regarding the values of anti-HIV-1 antibodies, viral load, T CD4^+^ lymphocyte count, stage of disease according to CDC classification criteria, age at diagnosis of AIDS, laboratory investigations, therapy used, date of start and discontinuation of antiretroviral therapy, start and type of *Pneumocystis jirovecii* pneumonia prophylaxis.

Through the use of reporting forms, the referral physician of each center in the Registry provides to transfer to the two coordination centers basic information regarding those who have acquired HIV-1 infection within 13 years of life or who are at risk of becoming infected because they were born to HIV-1 positive mothers; while the follow-up forms are filled in and updated every six months by a paediatrician in charge in each center. In fact, once an infection has been diagnosed, the children are visited every two months. In addition, any changes in clinical and immunological status, together with vaccinations, tests performed and treatment used, are recorded on the follow-up form. Children born to HIV-1 positive mothers who have not acquired the infection are examined at least once a year. Through a double cross-check in the two coordination centers, the quality of the information forwarded is checked. In addition, other control mechanisms are used, while maintaining the anonymity of children, to avoid double reporting. Finally, at least once a year, an audit of the referring physicians is organized in order to verify the results obtained, to plan and encourage research lines and to standardize procedures.

At the moment of patient’s enrolment, written informed consent is obtained from a patient’s parent or legal guardian; data are treated anonymously as each patient is identified by an alphanumerical code. The study was approved by the review boards and ethics committees of each participating institution; in particular, the ultimate form of the study was approved by the Ethics Committee for Human Investigation at Meyer Children’s University Hospital.

### Study design

We performed a cross-sectional study. Children enrolled into the *Italian Register for HIV Infection in Paediatrics* were selected whether they: have perinatal HIV-1 infection, were receiving cART and were in follow-up at the December 31, 2018. Children were excluded from the study whether they were: uninfected; with horizontally acquired HIV-1 infection (through transfusions, drug addiction, other); not receiving cART at the December 31, 2018, or aged > 18 years.

The following information has been collected for each patient: age (years); sex (male/female); body weight (Kg); clinical class (class N, A, B, C according to CDC classification); immunological category (category 1, 2, 3 according to CDC classification); CD4^+^ T lymphocytes count (cell/μL); birthplace of the child and his/her mother (Italian nationality/immigrant), viral load (VL, copies/mL).

The therapy children were receiving was categorized by distinguishing between off-label and on-label use. We have considered off-label use when the drug was prescribed under the age or weight it was licensed for according to the European Medicine Agency (EMA) [5]. Unfortunately, due to a large portion of missing data regarding the children’s weight, it was possible to evaluate off-label prescription by weight only relatively to few drugs.

For each therapeutic regimen the following data have been extracted from the Register: type of therapeutic regimen, number of drugs and specific drugs included in the regimen, year in which the therapeutic regimen was started, duration of therapy, age at the beginning of the regimen, clinical status, viral load and count and percentage of CD4^+^T lymphocytes at the beginning of therapy and at the last follow-up and the age at the last control and reported adverse events.

Immunological success was defined as a percentage of CD4^+^T lymphocytes > 25% or an increase > 10% between the beginning of the regimen and the last observation if the regimen was in progress or at the end of the regimen if it was discontinued. When the percentage of CD4^+^ T lymphocytes at the baseline and the last observations were both > 25%, we considered it as an immunological success. Virological success was defined as a VL not detectable at the last observation during therapy. Due to changes in the detection limits of the HIV RNA test over time, the undetectable VL was defined as < 400 copies/mL.

### Definitions

#### Definition of perinatal HIV-1 infection:

Perinatal infection is defined as the transmission of the infection in utero (congenital infection), during labour, or immediately after birth in breastfed children [8].

HIV-1 infection was diagnosed on the basis of the persistence of anti-HIV-1 antibodies after 18 months of life or, before 18 months, by detecting the positivity of viral markers on at least two occasions, excluding any investigations carried out on umbilical cord blood. Children for whom the detection of antibodies to HIV-1 and the PCR investigation were negative on at least two occasions are defined as non-infected [9].

#### Definition of cART

cART was defined as the combination of three or more antiretroviral drugs of at least two different classes [10, 11].

#### Definition of off-label use of drugs in paediatric age

Off-label use of a drug was defined as the use of pharmaceutical drugs for an unapproved indication or in an unapproved age group, dosage, or route of administration, according to the package leaflet of medicines authorized by EMA [5]. Detailed data are reported in Additional file 1: Table S1.

### Statistical analysis

The continuous variables were expressed as median and interquartile range (IQR). The categorical variables have been expressed as numbers and percentages.

For the categorical variables we performed the χ^2^ or Fisher test; for the continuous variables the medians between groups were compared using the Wilcoxon Mann–Whitney U test. Univariate logistic regression analysis was used to evaluate possible risk factors for receiving an off-label therapy, and correspondent odds ratios (ORs) and 95% confidence intervals were calculated. Statistical analyses were performed using the STATA/SE version 10.0 software package (Stata Corporation, College Station, TX). p < 0.05 was considered statistically significant.

## Results

Two hundred and twenty-five children were included in the study. Characteristics of the study children are summarized in Table 1.

**Table 1:** Characteristics of the included children (N = 225)

	Children on label antiretroviral treatment (n)			Children off-label antiretroviral treatment (n)			Total (n)	Total (%)	*p* value
All children	175 (77.7%)	% on label	% (within category)	50 (22.2%)	% off label	% (within category)	225	100%	
Sex									
Males	81	46.2%	77.88%	23	46.0%	22.12%	104	100%	
Females	94	53.8%	77.69%	27	54.0%	22.31%	121	100%	0.97
Age last controls									
< 1 year	0	0.0%	0.00%	2	4.0%	100.00%	2	100%	
1–2 years	7	4.0%	87.50%	1	2.0%	12.50%	8	100%	
3–5 years	16	9.1%	84.21%	3	6.0%	15.79%	19	100%	
6–11 years	47	26.9%	70.15%	20	40.0%	29.85%	67	100%	
≥ 12 years	105	60.0%	81.40%	24	48.0%	18.60%	129	100%	
Median age (months; IQR)	169.13 (106–204.9)			137.08 (98.5–181.5)			159.33 (102.6–201.2)		0.0519
Follow-up									
Followed since birth	52	29.8%	78.79%	14	28.0%	21.21%	66	100%	0.814
Origin (Child/Mother)									
Foreign Country/Missing	22	12.6%	91.67%	2	4.0%	8.33%	24	100%	
Foreign Country/Foreign Country	54	30.9%	80.60%	13	26.0%	19.40%	67	100%	
Italy/Missing	33	18.9%	80.49%	8	16.0%	19.51%	41	100%	
Italy/Foreign Country	42	24.0%	76.36%	13	26.0%	23.64%	55	100%	
Italy/Italy	24	13.8%	63.16%	14	28.0%	36.84%	38	100%	
Age at the beginning of cART									
< 1 year	7	4.0%	58.33%	5	10.0%	41.67%	12	100%	
1–2 years	14	8.0%	56.00%	11	22.0%	44.00%	25	100%	
3–5 years	23	13.1%	82.14%	5	10.0%	17.86%	28	100%	
6–11 years	44	25.1%	72.13%	17	34.0%	27.87%	61	100%	
≥ 12 years	87	49.8%	87.88%	12	24.0%	12.12%	99	100%	
Median age (months, IQR)	135 (65.4–184.27)			97.63 (17.7- 136.7)			126.6 (56.3–176.67)		0.001
Number of previous cART regimens									
1 regimen	30	17.1%	66.67%	15	30.0%	33.33%	45	100%	0.045
2 regimens	43	24.5%	86.00%	7	14.0%	14.00%	50	100%	
3 regimens	28	16.0%	71.79%	11	22.0%	28.21%	39	100%	
4 regimens	20	11.4%	74.07%	7	14.0%	25.93%	27	100%	
5 regimens	22	12.6%	81.48%	5	10.0%	18.52%	27	100%	
6 regimens	16	9.1%	94.12%	1	2.0%	5.88%	17	100%	
7 regimens	7	4.0%	77.78%	2	4.0%	22.22%	9	100%	
8 regimens	5	2.8%	83.33%	1	2.0%	16.67%	6	100%	
9 regimens	1	0.6%	100.00%	0	0.0%	0.00%	1	100%	
10 regimens	1	0.6%	50.00%	1	2.0%	50.00%	2	100%	
11 regimens	2	1.1%	100.00%	0	0.0%	0.00%	2	100%	
Pre-cART VL									
Detectable	52	29.8%	68.42%	24	48.0%	31.58%	76	100%	0.021
Undetectable	68	38.9%	83.95%	13	26.0%	16.05%	81	100%	
Missing	55	31.4%	80.88%	13	26.0%	19.12%	68	100%	
VL at last check									
Detectable	29	16.6%	74.36%	10	20.0%	25.64%	39	100%	0.583
Undetectable	145	82.9%	78.38%	40	80.0%	21.62%	185	100%	
Missing	1	0.6%	100.00%	0	0.0%	0.00%	1	100%	
Pre cART CD4^+^ lymphocytes count									
< 15%	12	6.9%	85.71%	2	4.0%	14.29%	14	100%	0.472
15–24%	15	8.6%	75.00%	5	10.0%	25.00%	20	100%	
≥ 25%	91	52.0%	77.78%	26	52.0%	22.22%	117	100%	
Missing	57	32.6%	77.03%	17	34.0%	22.97%	74	100%	
CD4^+^ lymphocytes count at last check									
< 15%	4	2.3%	50.00%	4	8.0%	50.00%	8	100%	0.0543
15–24%	15	8.6%	93.75%	1	2.0%	6.25%	16	100%	
≥ 25%	156	89.1%	77.61%	45	90.0%	22.39%	201	100%	

*VL* viral load, *cART* combined antiretroviral therapy

175/225 children (77.7%) were receiving on-label cART, while 50/225 (22.2%) were receiving an off-label regimen. At last check, off-label cART was administered to the 100% (2/2) of children aged < 1 years, 12.5% (1/8) of children aged 1–2 years, 15.8% (3/19) between 3 and 5 years, 29.9% (20/67) between 6 and 11 years, and 18.6% (24/129) > 12 years.

VL prior of current cART therapy was detectable in 48.0% (24/50) of off-label patients and in 29.7% (52/175) of on-label patients (p = 0.021), while no difference among groups was observed ad last check (detectable VL in 20.0% of children receiving off label cART and in 16.6% of children on on-label cART; p = 0.583) (Table 1).

Proportions of children with a CD4^+^ T lymphocytes percentage < 15% pre-current cART and at last check were similar in on-label and off-label patients (Table 1).

At univariate analysis, risk factors significantly associated with the probability to receive off-label cART were a pre-therapy detectable VL (OR: 2.41; 95%IC 1.13–5.19; p = 0.024), age < 2 years at the beginning of the current regimen (OR: 3.24; 95% CI 1.63–7.30; p = 0.001) and being on the first cART regimen (OR: 2.07; 95% CI 1.0006–4.26; p = 0.048) (Table 2).

**Table 2:** Univariate logistic regression analysis reporting odds ratios, 95% confidence intervals and p values of factors possibly associated with risk of receiving  off-label antiretroviral therapy

	Children receiving off-label cART (n/N)	%	OR	95% IC	p-value
Age at last check					
< 2 years	3/10	30.0%	1		
≥ 2 years	47/215	21.9%	1.53	(0.38–6.15)	0.548
Sex					
Males	23/104	22.1%	1		
Females	27/121	22.3%	0.988	(0.52–1.85)	0.971
Origin					
Foreign Country	15/91	16.5%	1		
Italy	35/134	26.1%	1.791	(0.91–3.51)	0.090
Age at the Beginning of the current regimen					
≥ 2 years	34/188	18.1%	1		
< 2 years	16/37	43.2%	3.24	(1.63–7.30)	0.001
Nr. of previous cART regimens					
< 1	15/45	33.3%	1		
1	35/180	19.4%	207	(1.0006–4.26)	0.048
Pre-cART VL^*^					
Undetectable	13/81	16.0%	1		
Detectable	24/76	31.6%	2.414	(1.13–5.19)	0.024
Missing	13/68	19.1%	1.236	(0.53–2.88)	0.623
VL^*^ at last check					
Undetectable	40/185	21.6%	1		
Detectable	10/39	25.6%	1.25	(0.56–2.78)	0.584
Missing	0/1	0.0%	omitted		
Pre-cART CD4^+^ lymphocytes count					
< 15	2/14	14.3%	1		
> 15	31/137	22.6%	1.755	(0.37–8.26)	0.477
Missing	17/74	23.0%	1.789	(0.36–8.79)	0.474
CD4^+^ lymphocytes percentage at last check					
< 15%	4/8	50.0%	1		
> 15%	46/217	21.2%	0.269	(0.06–1.12)	0.071

*VL* viral load, *cART* combined antiretroviral therapy

At multivariate analysis age < 2 years was confirmed to be an independent risk factor for receiving off-label cART (OR: 2.75; 95% CI 1.24–6.09; p = 0.013) (Table 3).

**Table 3:** Multivariate logistic regression analysis reporting odds ratios, 95% confidence intervals and p values of factors possibly associated with risk of receiving  off-label antiretroviral therapy

	Children receiving off-label cART (n/N)	%	OR	95% IC	p-value
Age at the Beginning of the current regimen					
≥ 2 years	34/188	18.1%	1		
< 2 years	16/37	43.2%	2.75	(1.24–6.09)	0.013
Pre-cART VL^*^					
Undetectable	13/81	16.0%	1		
Detectable	24/76	31.6%	2.078	(0.95–4.56)	0.068
Missing	13/68	19.1%	1.103	(0.47–2.61)	0.824

*VL* viral load, *cART* combined antiretroviral therapy

### Sub-analysis of most commonly drugs prescribed off-label

The most commonly off-label drugs administered were: lopinavir/ritonavir (10/56, 17.9%) tenofovir disoproxil/emtricitabine (11/11, 100%), dolutegravir/abacavir/lamivudine (8/26, 30.8%), elivitergravir/cobicistat/emtricitabine/tenofovir alafenamide (5/11, 45.5%) (Table 4).

**Table 4:** Percentages of combined antiretroviral drugs prescribed off-label by children’s age and weight

		Antiretroviral drugs prescribed off label by age				Antiretroviral drugs prescribed off label by weight		
	On label (n)	Off label (n)	Total	Off label (%)	On label	Off label (n)	Missing	Total
Nucleoside Reverse Transcriptase Inhibitors (NRTI)								
Abacavir (ABC)	35	1	36	2.8%	26	0	10	36
Didanosina (ddI)	1	0	1	0.0%	0	0	1	1
Emtricitabina (FTC)	4	0	4	0.0%	2	0	2	4
Lamivudina (3TC)	76	5	81	6.2%	44	0	37	81
Tenofovir (TDF)	4	0	4	0.0%	3	0	1	4
Zidovudina (ZDV)	26	0	26	0.0%	15	0	11	26
Tenofovir alafenamide (TAF)	3	0	3	0.0%	1	0	2	3
Non-nucleoside reverse transcriptase inhibitors (NNRTI)								
Efavirenz (EFV)	8	1	9	11.1%	4	0	5	9
Etravirina (EVR)	3	2	5	40.0%	3	2	0	5
Nevirapina (NVP)	19	0	19	0.0%	11	0	8	19
Rilpivirina (RPV)	1	0	1	0.0%	0	0	1	1
Protease inhibitors (PI)								
Atazanavir (ATV)	2	2	4	50.0%	3	0	1	4
Darunavir (DRV)	46	4	50	8.0%	30	1	19	50
Ritonavir (RTV)	51	1	52	1.9%	32	0	20	52
Integrase inhibitors (INSTI)								
Raltegravir (RAL)	21	1	22	4.5%	20	0	2	22
Dolutegravir (DTG)	18	0	18	0.0%	7	0	11	18
Elvitegravir (EVG)	2	0	2	0.0%	2	0	0	2
Booster								
Cobicistat	1	0	1	0.0%	1	0	0	1
Fixed-dose combined drugs								
Lopinavir/ritonavir	46	10	56	17.9%	39	0	17	56
Zidovudine/lamivudine	7	1	8	12.5%	5	1	2	8
Lamivudine/abacavir	30	0	30	0.0%	18	3	9	30
Tenofovir/emtricabine	0	11	11	100.0%	6	5	0	11
Emtricitabine/tenofovir/efavirenz	0	1	1	100.0%	1	0	0	1
Emtricitabina/rilpivirina/tenofovir	0	1	1	100.0%	1	0	0	1
Dolutegravir/abacavir/lamivudine	18	8	26	30.8%	15	5	6	26
Darunavir/cobicistat	11	0	11	0.0%	4	0	7	11
Tenofovir alafenamide/emtricitabine	15	2	17	11.8%	11	1	5	17
Elvitegravir/cobicistat/emtricitabine/tenofovir alafenamide	6	5	11	45.5%	7	4	0	11
Emtricitabine/rilpivirine/tenofovir alafenamide	7	0	7	0.0%	3	0	4	7
Darunavir/cobicistat/emtricitabine/tenofovir alafenamide	5	0	5	0.0%	5	0	0	5

VL at last check, in patients treated with lopinavir/ritonavir, was undetectable in 90% (9/10) of off-label patients and in 82.2% (37/45) of on-label patients (*p* = 0.547), whereas the last CD4^+^ T lymphocytes percentage was > 25% in 100% (10/10) of off-label patients and 91.1% (41/45) of on-label patients (*p* = 0.983) (Additional file 1: Table S2).

Regarding emtricitabine/tenofovir disoproxil, at last check VL was undetectable in 81.8% (9/11) of off-label patients, whereas the last CD4^+^ T lymphocytes percentage was > 25% in 81.8% (9/11) of off-label patients. We also observed that no child received on-label treatment with this drug (Additional file 1: Table S3).

Regarding elvitegravir/cobicistat/emtricitabine/tenofovir alafenamide, at last check the VL was undetectable in 100% (5/5) of off-label patients and in 100% (6/6) of on-label patients, whereas the last CD4^+^ T lymphocytes percentage was > 25% in 100% (5/5) of off-label patients and in 100% (6/6) of on-label patients (Additional file 1: Table S4).

Finally, at last check VL in patients on therapy with abacavir/dolutegravir/lamivudine was undetectable in 87.5% (7/8) of off-label patients and in 77.7% (14/18) of on-label patients (*p* = 0.561), whereas the last CD4^+^ T lymphocytes percentage was > 25% in 87.5% (7/8) of off-label patients and in 94.4% (17/18) of on label patients (*p* = 0.539) (Additional file 1: Table S5).

## Discussion

Off-label drugs in our cohort of perinatally HIV-1 infected children were prescribed to 22.2% (50/225) of children at their last check in 2018. At univariate logistic regression analysis, risk factors for receiving an off-label regimen were age < 2 years, being of first HAART regimen and a detectable VL before starting that regimen. At multivariate analysis only younger age was confirmed to be an independent risk factor for receiving an off-label therapy. However, we also noticed that several children (56.52%, 13/23), aged between 11 and 12 years of age were receiving an off-label cART at last check. In this sub-group of children physicians probably considered minimal the potential risks related to an off-label use of the ARV drug since 12 years was the age for which the drug was licensed.

The most commonly antiretroviral drugs prescribed off -label were lopinavir-ritonavir (Kaletra^®^) in younger children, and three fixed dose combinations in older children: elvitegravir/cobicistat/emtricitabine/tenofovir alafenamide (Genvoya^®^), abacavir/dolutegravir/lamivudine (Triumeq^®^) and tenofovir disoproxil/emtricitabine (Truvada^®^).

Comparing our results to a 2016 Spanish multicentre study, conducted by Cooke et al. on a cohort of 318 perinatally HIV-1-infected children and adolescents between 1988 and 2012, it was found that our off-label therapy rates in children are lower than the ones of the Spanish sample, which reveals an off-label use of antiretroviral therapy in 69% of included children (221/318) [6]. This difference could reflect the increase in approval coverage for the clusters under 2 years of age, which occurred after 2012 and consequently reduced the off-label incidence. For example, in 2012 atazanavir was approved in children over 6 years of age, while in 2014 the approval was brought forward to children over 3 months of age; raltegravir in 2012 was approved only in children over 2 years of age, while by 2018 it was approved from birth; efavirenz, which until 2013 was approved only for children over 3 years of age, is now approved in children over 3 months of age [5]. Considering that efavirenz was used by the 24.5% of our population and that among the non-nucleoside reverse transcriptase inhibitors (NNRTIs) it was the most widely used after nevirapine, this fact may have contributed to reduce the off-label percentage in our population. However, in our cohort, off-label use was still widespread. The 10% of our off-label patients have reached the fifth therapeutic regimen; this confirms that off-label therapies are chosen in some multi-experienced children, failing previous therapy and probably with no in-label therapeutic options available.

In our study, before the current cART, the 48% of the off-label children had a detectable VL, while, at the last check, the 80% of the patients had an undetectable VL and the 90% a CD4^+^ lymphocyteS count > 25%. Similar to our results, Cooke et al*.* found that the main reason for the off-label use of cART was the virological failure of the previous therapy. Moreover, the onset of adverse events and problems related to the administration of drugs, such as formulations unsuitable for age, respectively led in 12% and 5% of cases to the discontinuation of treatment. Adherence to the therapy is essential for its effectiveness and this is in agreement with our data, since the main drugs used off-label are fixed-combination drugs. Clay et al. meta-analysis, comparing adult patients’ adherence of single tablet regimens (STR) to multiple tablet regimens (MTR), revealed that patients on STR cART were significantly more adherent and had a better VL suppression when compared to patients on MTR [12]. Indeed, in our study, we observed that four MTR (lopinavir/ritonavir; emtricitabine/tenofovir disoproxil; elvitegravir/cobicistat/emtricitabine/tenofovir alafenamide and abacavir/dolutegravir/lamivudine) were the most commonly used off-label drug formulations.

Since 2012 lopinavir/ritonavir is licensed for the use in children aged > 14 days, while since 2002 it was licensed for children aged > 2 years. In our dataset, the age group receiving off-label lopinavir/ritonavir was mostly represented by children aged < 12 months. Previous studies found that lopinavir/ritonavir is more effective than nevirapine, which may be one of the reasons why physician decided to start this therapy [13]. However, since cases of lopinavir/ritonavir cardiotoxicity (complete heart block and cardiomyopathy) have been reported in infants, caution is recommended when using this drug [13]. No cardiac adverse event in young children was reported in our dataset. What we can observe instead, is a good therapeutic success, demonstrated by the fact that 100% of children receiving off label lopinavir/ritonavir had a CD4^+^ T lymphocytes percentage at last check-up > 25% and 90% had an undetectable viremia.

For what concerns elvitegravir/cobicistat/emtricitabine/tenofovir alafenamide, the age most involved by their off-label use was 8–11 years; in our cohort, 100% of off-label patients the last VL was undetectable and the CD4^+^ T lymphocytes percentage > 25%. In the same age group, we also find off-label use for abacavir/dolutegravir/lamivudine. In these children an undetectable VL at the last check was reached in the 87.7% of patients and undetectable and CD4^+^ T lymphocytes percentage > 25% in the 87.5% of them.

Regarding emtricitabine/tenofovir disoproxil, the age most involved by off-label use was 11–14 years, (the drug has to be considered off label when used in children aged 14 years since when these children started the therapy it was approved > 18 years). At the last check the VL was undetectable in the 81.8% and CD4^+^ T lymphocytes percentage > 25% in the 81.8%.

In our sample, a 10 years old child received an off-label treatment with rilpivirine, emtricitabine and tenofovir disoproxil. Before the current therapy the patient had a detectable viral load, while at the last check the VL was undetectable. No adverse event was reported for this patient. A previous multicentre study that enrolled 17 children and adolescents with perinatal HIV-1 infection from 2013 to 2015 demonstrated the efficacy of off-label use of rilpivirine in combination with emtricitabine and tenofovir disoproxil, as children showed good control of the infection, improved CD4^+^ T lymphocytes count and CD4^+^/CD8^+^ T lymphocytes ratio [14].

Our data suggest similar proportion of virological and immunological success at last check among children receiving off- or on-label cART. No adverse event to off-label ARV drug was reported in our dataset.

The potential limitations of our study lie in its retrospective design. In addition, one limitation of the study is the scarcity of data regarding the collected adverse events. Even if no adverse event related to the use of off-label ARV drug was reported we may not exclude an underreporting bias. Other studies are needed to better elucidate this issue. Another possible limitation of our study is that in our dataset young children (aged less than 2 years) were more likely to receive off label drugs than older patients. However, the great majority of young children received lopinar/ritonavir, which has been subsequently licenced for the use in children aged 14 days or more. More recent studies, considering recent updates in paediatric drug licences, might lead to a different conclusion. Similarly, since patients’ information was collected up to 2018, a trend toward a general reduced off label use of ARV drugs is likely in recent years since new approvals have occurred over time.

Finally, we could not explore the risk of prescription of not-corrected dose in children receiving an off-label regimen, due to a large proportion of missing data regarding body weights.

In a previous study more than 10% of children treated with an off-label prescription received an overdose and a further 10% received an underdose, defined as the administration of a 25% dose respectively above or below the recommended dose [6]. A previous multi-centre cohort study from Ireland and UK, including 615 children aged 2–12 years and treated with cART, showed that children receiving a dose less than 90% of the recommended dose accounted for 6–62% of the study population [15].

## Conclusions

The prescription of an off-label cART in perinatally HIV-1 infected children is common, in particular in children with detectable VL despite previous therapies. Our data suggest similar proportion of virological and immunological success at last check among children receiving off- or on-label cART. Even if our safety data regarding off-label regimens where poor, no adverse event was reported in our dataset. However, larger studies are needed to better clarified efficacy and safety of off-label cART regimens in children.

In our dataset, the prescription of an off-label cART was more common in children aged < 2 years, likely due to the fact that only the 34.4% of ARVs are approved for use in this age group. Moreover, we could assume that in several cases the physician choice to undertake an off-label therapy was probably dictated by a virological failure with previous regimens, possibly due to the presence of drug resistance viral strains or poor patient adherence.

Efforts should be made in order to allow an on-label prescription in HIV-1 infected children.

## Supplementary Information

**Additional file 1. Table S1**. Antiretroviral drugs for children and adolescents according to European Medicines Agency; quaque die (qd), bis in die (bid), quater in die (qid), tablet (cp), prolonged release (RP).** Table S2**. Characteristics of children receiving lopinavir/ritonavir off-label.** Table S3**. Characteristics of children receiving emtricitabine/ tenofovir disoproxil off-label (no child received on-label treatment in this group).** Table S4**. Characteristics of children receiving elvitegravir/cobicistat/emtricitabine/tenofovir alafenamide off-label.** Table S5**. Characteristics of children receiving abacavir/dolutegravir/lamivudine off-label.

## Data Availability

The datasets used and analysed during the current study are available from the corresponding author on reasonable request.

## Abbreviations

HAART: Highly active antiretroviral therapy
EMA: European medicine agency
VL: Viral load
ARV: Antiretroviral
cART: Combination antiretroviral therapy
IQR: Interquartile range
NNRTIs: Non-nucleoside reverse transcriptase inhibitors
STR: Single tablet regimens
MTR: Multiple tablet regimens
