# Supplementary material for: Off-label use of combined antiretroviral therapy, analysis of data collected by the Italian Register for HIV-1 infection in paediatrics in a large cohort of children
Source: BMC Infect Dis. 2022 Jan 15;22:55. doi: 10.1186/s12879-022-07026-w (PMC8760752; doi:10.1186/s12879-022-07026-w)
Supplement: Supplementary file 1 — Additional file 1. Table S1. Antiretroviral drugs for children and adolescents according to European Medicines Agency; quaque die (qd), bis in die (bid), quater in die (qid), tablet (cp), prolonged release (RP). Table S2. Characteristics of children receiving lopinavir/ritonavir off-label. Table S3. Characteristics of children receiving emtricitabine/ tenofovir disoproxil off-label (no child received on-label treatment in this group). Table S4. Characteristics of children receiving elvitegravir/cobicistat/emtricitabine/tenofovir alafenamide off-label. Table S5. Characteristics of children receiving abacavir/dolutegravir/lamivudine off-label. [file 12879_2022_7026_MOESM1_ESM.docx]

**Table S1. Antiretroviral drugs for children and adolescents according to European Medicines Agency; quaque die (qd), bis in die (bid), quater in die (qid), tablet (cp), prolonged release (RP).**

| DRUGS | AGE | WEIGHT | DOSE mg/kg | NUMBER OF ADMINISTRATIONS | TYPE OF FORMULATION | | | | | | APPROVAL DATE | | | DOSE |
| --- | --- | --- | --- | --- | --- | --- | --- | --- | --- | --- | --- | --- | --- | --- |
| **Abacavir (ABC)** | ≥ 3 months  (EMA) | any | <25Kg 16mg/kg ~ >25 kg 600mg | 1 or 2 doses if divided | Oral solution (20 mg/ml) | | | Film-coated tablets: (300 mg) | | | n^o^ | date | age | • from 14 kg to <20 kg: **300 mg 1 tablet once daily** • from≥20 kg to <25 kg: **450 mg Qnce daily (1tbe ½tb)** • ≥25 kg: **600 mg 1 tablet once daily** |
|  |  |  |  |  | age | kg | | age | | kg | 2 | 08/07/1999 | adults |  |
|  |  |  |  |  | ≥3months | < 14 | | ≥ 3 months | | ≥ 14 |  | 24/10/2001 | 3 months |  |
| **Emtricitabine (FTC)** | ≥ 4 months  (EMA) | any | 6 mg/kg dose maximum 240 mg (24 ml) | 1 | Oral solution (10 mg/ml) | | | Tablet  (200 mg) | | | n^o^ | date | age | Adults: 200mg Once daily |
|  |  |  |  |  | age | kg | | age | | kg | 2 | 24/10/2003 | adults |  |
|  |  |  |  |  | ≥ 4months | < 33 | | ≥ 4 months | | ≥ 33 |  | 24/10/2004 | 4 minths |  |
| **Lamivudine (3TC)** | ≥ 3 months  (EMA) | • Oral solution **< 14 kg**  • Film-coated tablets **≥ 14 kg** | <12 years 4mg/kg  Oral solution:10mg/ml >12 years: 300mg | one or two doses if the dose is divided in half | Oral solution (10 mg/ml) | | | Film-coated tablets: (150mg  300mg) | | | n^o^ | date | age | • from 14 kg to <20 kg: **150 mg Once daily**  • from ≥20 kg tp <25 kg: **225 mg/day (tb of 150 mg morning and 1tb of 150 mg evenig, or 225 mg once daily)** • ≥25 kg: **300 mg/day (2tb of 150 mg or 1 tb of 300 mg)** |
|  |  |  |  |  | age | kg | | age | | kg | 2 | 08/08/1996 | adults |  |
|  |  |  |  |  | ≥3 months | < 14 | | ≥ 3 months | | ≥ 14 |  | 08/08/1996 | 3 months |  |
| **Tenofovir Disoproxil Fumarate (TDF)** | ≥ 2 years (EMA) | • tablets **≥ 17 kg** | 8mg/kg ~ | 1 | Granules for Oral Suspension: (40 mg/1g) | | | Film-coated tablets: (150mg  200mg 250mg) | | | n^o^ | date | age | • da 17 kg a <22 kg: **150 mg Once Daily** • da ≥22 kg a <28 kg: **200 mg Once Daily** • da ≥28 kg a <35 kg: **250 mg Once Daily** • ≥ 35 kg: **300 mg Once Daily** |
|  |  |  |  |  | age | kg | | age | | kg | 2 | 05/02/2002 | adults |  |
|  |  |  |  |  | ≥ 2 years | --- | | ≥2 years | | ≥ 17 |  | 22/11/2012 | 2 years |  |
| **Tenofovir Alafenamide (TAF)** | ≥ 12 years (EMA) | • **≥ 35 kg** | • F/TAF 200/25 mg • E/C/F/TAF 150/150/200/10 mg • F/TAF/RPV 200/25/25 mg | 1 | Tablet  (10 mg 25 mg) | | | | | | n^o^ | date | age | • **F/TAF 200/25 mg Once Daily** • **E/C/F/TAF 150/150/200/10 mg Once Daily** • **F/TAF/RPV 200/25/25 mg Once Daily** |
|  |  |  |  |  | age | | | kg | | | 1 | 09/01/2017 | >12 years |  |
|  |  |  |  |  | ≥ 12 years | | | ≥ 35 | | |  |  |  |  |
| **Zidovudine (AZT)** | since birth (EMA) | any | newborn child:2mg/kg   ≥4 kg a <9 kg: 12 mg/kg (1,2 ml/kg) Twice-Daily   ≥9 kg a <30 kg: 9 mg/kg (0,9 ml/kg) Twice-Daily  ≥30 kg: 300 mg Twice-Daily | 4   2  2   2 | Oral Syrup (10mg/ml) | | solution for infusion  (10 mg/ml fiala da 20 ml) | | Capsule (100 mg 250 mg) | | n^o^ | date | age | • since birth to < 4 kg: **2 mg/kg four Daily** • da ≥4 kg to <9 kg: **12 mg/kg (1,2 ml/kg) twice daily**  • da ≥9 kg to <30 kg: **9 mg/kg (0,9 ml/kg) twice daily**  • ≥30 kg: **300 mg twice daily**  • from 8 kg to 13 kg: **200 mg/day(1tb da 100 mg twice daily )** • from14 kg to 21 kg: **300 mg/day (1tb of 100 mg morning and 2tb of 100 mg evening)** • from 22 kg to 30 kg: **400 mg/day (2tb of 100 mg morning and 2tb of 100 mg evening)** • ≥ 30 kg: **300 mg twice daily** |
|  |  |  |  |  | età | kg | età | kg | età | kg | 2 | 19/03/1987 | adults |  |
|  |  |  |  |  | birth | --- | birth | --- | birth | ≥ 8 |  | 18/05/1994 | since birth |  |
| **ABC + 3TC** |  | • **≥ 25 kg** | 600mg ABC/300mg 3TC | 1 | Film-coated tablets: (600mg ABC/300mg 3TC) | | | | | | n^o^ | date | età | • **1tb Once Daily** |
|  |  |  |  |  | age | | | kg | | | 2 | 17/12/2004 | adults |  |
|  |  |  |  |  | ≥ 12 years | | | ≥ 25kg | | |  | 17/12/2004 | >12 years |  |
| **AZT + 3TC** | … | • **≥ 14 kg** | • form 14 kg to <21 kg: ½ tb Twice-Daily • from ≥21 kg to <30 kg: ½ tb morning e 1 tb evening • ≥30 kg: 1 tb Twice-Daily | 2 | Tablet (300mg AZT/150mg 3TC) | | | | | | n^o^ | date | age/weight | • from 14 kg to <21 kg: **½ tb twice daily**  • from ≥21 kg to <30 kg: **½ tb morning and 1 tb evening** • ≥30 kg: **1 tb twice daily** |
|  |  |  |  |  | age | | | kg | | | 2 | 18/03/1998 | 30kg |  |
|  |  |  |  |  | --- | | | ≥ 14 | | |  | 13/11/2007 | 14kg |  |
| **ABC + 3TC + AZT** | ≥ 18 years (EMA) | • **≥ 40 kg** | 300 mg (ABC), 150 mg (3TC), 300 mg (AZT) | 2 | Tablet (300mg ABC/150mg 3TC/300mg AZT) | | | | | | n^o^ | date | age/weight | • **1 tb twice daily** |
|  |  |  |  |  | age | | | kg | | | 1 | 28/12/2000 | 40kg |  |
|  |  |  |  |  | ≥ 18 years | | | ≥ 40 | | |  |  |  |  |
| **FTC + TDF** | ≥ 12 years (EMA) | • **≥ 35 kg** | 200 mg (FTC), 245 mg (TDF) | 1 | Tablet (200mg FTC/245mg TDF) | | | | | | n^o^ | date | age | • **1 tb Once Daily** |
|  |  |  |  |  | age | | | kg | | | 1 | 21/02/2005  16/12/2016 | adults >12 years |  |
|  |  |  |  |  | ≥ 12 years | | | ≥ 35 | | |  |  |  |  |
| **FTC + TAF** | ≥ 12 years (EMA) | • **≥ 35 kg** | • 200/25 mg con DTG, EFV, MCV, NVP, RVP, RAL  • 200/10 mg con DRV/r, DRV/c, ATV/r, ATV/C, LPV/r | 1 | Tablet (200mg FTC/25mg TAF) (200mg FTC /10MG TAF) | | | | | | n^o^ | date | age | • **200/25 mg with DTG, EFV, MCV, NVP, RVP, RAL 1tb Once daily**  • **200/10 mg with DRV/r, DRV/c, ATV/r, ATV/C, LPV/r 1tb Once daily** |
|  |  |  |  |  | age | | | kg | | | 2 | 24/04/2016 | >12 years |  |
|  |  |  |  |  | ≥ 12 years | | | ≥ 35 | | |  |  |  |  |
| **Efavirenz (EFV)** | ≥ 3 years (EMA) | • **≥ 3,5 kg** | • 3,5 kg to <5kg: 1 tb 100 mg once daily  • ≥5 kg to<7,5 kg: 1 tb da 100 mg + 1 cp 50 mgonce daily  • ≥7,5 kg to <15 kg: 1 tb da 200 mg once daily  • ≥15 kg to <20 kg: 1 tb da 200 mg + 1 cp da 50mg once daily | 1 | Capsule (50mg 100mg 200mg) | | | Tablet coated  (600mg) | | | n^o^ | date | age | • from 3,5 kg to <5kg: **1tb of 100 mg Once daily** • from ≥5 kg to <7,5 kg: **1 tb of 100 mg + 1 tb 50 mg Once daily** • from ≥7,5 kg to<15 kg: **1tb of 200 mg Once daily** • from≥15 kg to <20 kg: **1 tb of 200 mg + 1 tb og 50 mg Once daily** • from ≥20 kg to <25 kg: **3 tb of100 mg Once daily** • from ≥25 kg to <32,5 kg: **3 tb of 100 mg + 1 tb of 50 mg Once daily** • from ≥32,5 kg to <40 kg: **2 200 mg Once daily** • ≥ 40 kg: **1 tb of 600 mg o 3 tb of 200 mg Once daily** |
|  |  |  |  |  | age | kg | | age | | kg | 3 | 28/05/1999 | adults |  |
|  |  |  |  |  | ≥ 3 months | 3,5 | | ≥ 3months | | ≥ 3,5 |  | 28/05/1999 26/02/2015 | 3 years  3 months |  |
| **Etravirine (ETR)** | ≥ 6 years (EMA) | • **≥ 16 kg** | • 16 kg to <20kg: 100 mg (4 tb da 25 mg Twice-Daily or 1 tb 100 mg Twice-Daily) • ≥20 kg to <25 kg: 125 mg (5 cp da 25 mg Twice-Daily o 1 tb 100 mg + 1 tb da 25 mg Twice-Daily) | 2 | Tablet  (25mg 100mg 200mg) | | | | | | n^o^ | date | age | Age between 6 and 8 years: • from 16 kg to <20kg: **100 mg twice daily (4 cp da 25 mg twice daily or 1 tb of 100 mg twice daily** • from ≥20 kg to <25 kg: **125 mg twice daily (5 tb of 25 mg twice daily or 1tb of 100 mg + 1 tb of 25 mg twice daily)** • from ≥25 kg to <30 kg: 150 **mg twice daily (6 tb of 25 mg twice daily or  1 tb of 100 mg + 2 tb of 25 mg twice daily)** • ≥30 kg: **200 mg twice daily (8 tb of 25 mg twice daily or 2 tb of 100 mg twice daily o 1 tb of 200 mg twice daily) Aged 18 and over:** • **1 tb of 200 mg o 2 tb of 100 mg twice daily** |
|  |  |  |  |  | age | | | kg | | | 2 | 28/08/2008 | adults |  |
|  |  |  |  |  | ≥ 6 years | | | ≥ 16 | | |  | 06/03/2013 | >6 years |  |
| **Nevirapine (NVP)** | since birth (EMA) | • any | • Tablets:  150-200 mg/m2 Once daily for 14 days (max 200 mg/day), after 150-200 mg/m2 Twice-Daily (max 400 mg/die) • Tablet coated : ≥ 6 years (0,58-0,83 m2) 200 mg Once Daily | 1 | Tablet  (200 mg) | | Tablet RP (50 mg 100 mg 400 mg) | | Suspension: 10 mg/ml) | | n^o^ | date | age | • Tablets:  **150-200 mg/m^2^ once dayli for 14 days (max 200 mg/day), after 150-200 mg/m^2^ twice daily (max 400 mg/day)** • Tablet extended-release: ≥ 6 years **(0,58-0,83 m^2^) 200 mg once daily (0,84-1,16 m^2^) 300 mg once daily (≥1,17 m^2^) 400 mg once daily** • Syrup:  **10 mg/ml** |
|  |  |  |  |  | age | kg | age | kg | age | kg | 2 | 05/02/1998 | adults |  |
|  |  |  |  |  | birth | --- | birth | --- | birth | --- |  | 18/06/1999 | since birth |  |
| **Rilpivirine (RPV)** | ≥ 12 years(EMA) | • any | 1 tb 25 mg Once Daily | 1 | Film-coated tablets:(25mg) | | | | | | n^o^ | date | age | • **1 tb of 25 mg once daily** |
|  |  |  |  |  | age | | | kg | | | 1 | 28/11/2011 | adults |  |
|  |  |  |  |  | ≥ 12 years | | | any | | |  |  |  |  |
| **EFV + FTC + TDF** | ≥ 12 years (EMA) | • **≥ 35 kg** | • 1tb 600 mg (EFV), 200 mg (FTC), 300 mg (TDF) | 1 | Film-coated tablets:  (600mg EFV/200mg FTC/300mg TDF) | | | | | | n^o^ | date | age | • **1 tb of 600 mg (EFV), 200 mg (FTC), 300 mg (TDF) once daily** |
|  |  |  |  |  | age | | | kg | | | 1 | 13/12/2007 | adults |  |
|  |  |  |  |  | ≥ 12 years | | | ≥ 40 | | |  | 09/11/2016 | >12 years |  |
| **FTC + RPV + TDF** | ≥ 18 years (EMA) | • any | • 1 tb 200 mg (FTC), 25 mg (RPV), 300 mg (TDF) Once Daily | 1 | Tablet  (200 mgFTC, 25 mg RPV, 300 mg TDF) | | | | | | n^o^ | date | age | • **1 tb of 200 mg (FTC), 25 mg (RPV), 300 mg (TDF) once daily** |
|  |  |  |  |  | age | | | kg | | | 1 | 28/11/2011 | adults |  |
|  |  |  |  |  | ≥ 12 years | | | ≥ 40 | | |  |  |  |  |
| **FTC + RPV + TAF** | ≥ 12 years (EMA) | • **≥ 35 kg** | • 1 tb 200 mg (FTC), 25 mg (RPV), 25 mg (TAF)  Once daily | 1 | Tablet  (200 mg FTC , 25 mg RPV, 25 mg TAF) | | | | | | n^o^ | date | age | • **1 tb of 200 mg (FTC), 25 mg (RPV), 25 mg (TAF) once daily** |
|  |  |  |  |  | age | | | kg | | | 1 | 21/12/2016 | >12 years |  |
|  |  |  |  |  | ≥ 12 years | | | ≥ 40 | | |  |  |  |  |
| **Atazanavir (ATV)** | ≥ 3 months  (EMA) | • **≥** **5 kg** | • from 5 kg to <15 kg: ATV/r 200/80 Once daily  • form ≥15 kg to 25 kg: ATV/r 250/80 mg  Once daily | 1 | Powder Packet: (50mg/1,5mg) | | | Capsule 150 mg 200mg 300mg | | | n° | date | age | age between 3 months and 6 years:powder packets  • from 5 kg to <15 kg: **ATV/r 200/80 once daily**  • from ≥15 kg to 25 kg: **ATV/r 250/80 mg once daily**    Age over 6 years:capsules • from 15 kg to <20 kg: **150 mg once daily + RTV 100 mg once daily**  • from ≥20 to <40 kg: **200 mg once daily + RTV 100 mg once daily**  • ≥40 kg: **300 mg once daily + RTV 100 mg once daily** |
|  |  |  |  |  | age | kg | | age | | kg | 3 | 02/03/2004 | adults |  |
|  |  |  |  |  | ≥ 3months | >5kg | | ≥ 6years | | ≥ 15kg |  | 05/07/2010 10/04/2014 | >6years  3 months |  |
| **Darunavir (DRV)** | ≥ 3 years (EMA) | **≥ 10 kg** | from 10 kg to <11 kg: 200 mg + RTV 32 mg   from ≥11 kg to <12 kg: 220 mg + RTV 32 mg  from ≥12 kg to <13 kg: 240 mg + RTV 40 mg Twice-Daily | 2 | Oral Suspension: (100mg/ml) | | | Tablets (75mg  150 mg 300mg 600mg 800mg) | | | n^o^ | date | age | • from10 kg to <11 kg: **200 mg twice daily + RTV 32 mg twice daily** • from ≥11 kg to <12 kg: **220 mg twice daily+ RTV 32 mg twice daily** • from≥12 kg to <13 kg: **240 mg twice daily+ RTV 40 mg twice daily** • from ≥13 kg to <14 kg: **260 mg twice daily+ RTV 40 mg twice daily** • from ≥14 kg to <15 kg: **280 mg twice daily+ RTV 48 mg twice daily** • from ≥15 kg to<30 kg: **380 mg twice daily + RTV 50 mg twice daily** • from≥30 kg to<40 kg: **460 mg twice daily + RTV 60 mg twice daily** • ≥40 kg: **600 mg twice daily + RTV 100 mg twice daily**  • from 15 kg a <30 kg: **375 mg twice daily+ RTV 50 mg twice daily** • from ≥30 kg a <40 kg: **450 mg twice daily + RTV 60 mg twice daily** • ≥40 kg: **600 mg BID + RTV 100 mg twice daily o 800 mg twice daily + RTV 100 mg twice dailyse "ART experienced", without DRV mutations** |
|  |  |  |  |  | age | kg | | age | | kg | 2 | 12/02/2007 | adults |  |
|  |  |  |  |  | ≥ 3 years | >10kg | | ≥ 3 years | | ≥ 15kg |  | 23/06/2009 | > 3 years |  |
| **Lopinavir/ritonavir (LPV/r)** | ≥ 14 days (EMA) | any | from 14gg to 6 months :16/4 mg/kgfrom 6 months to 18 years and <15kg 12/3 mg/kg between 15kg e 40kg 10/2,5 mg/kg 2 | 2 | Oral solution80/20mg/ml LPVRTV | | | Tablet 200 mgLPV+50mg RTV100 mg LPV+25mg RTV | | | n^o^ | date | age | from 14 days to 6 months:16/4 mg/kgfrom 6 months to 18 years and <15kg 12/3 mg/kg between 15kg e 40kg 10/2,5 mg/kg 2 twice daily |
|  |  |  |  |  | age | kg | | età | | kg | 3 | 20/03/2001 | adults |  |
|  |  |  |  |  | ≥ 14 days | --- | | ≥ 2 years | | --- |  | 20/03/2001 23/09/2012 | >2 years >14 days |  |
| **Ritonavir (RTV)** | ≥ 2 years (EMA) | unspecified | 350 mg/m² Adults :600mg | 2 | Oral solution (20mg/ml) | | | Tablet  100mg | | | n^o^ | date | age | • **1 tb da 100 mg**  Adults:600mg |
|  |  |  |  |  | age | kg | | age | | kg | #### | 26/08/1996 | adults |  |
|  |  |  |  |  | ≥ 2 years | --- | | ≥ 2 years | | --- |  | 26/02/1999 | 2 years |  |
| **Enfuvirtide (T-20)** | ≥ 6 anni (EMA) | • **≥ 11 kg** | 2mg/kg | 2 | Lyophilized Powder for Injection vials:108mg/1,1ml(90mg/1ml) | | | | | | n^o^ | date | age | 6 to 12 years old : 2 mg/kg twice dailys under skin (dose max 90 mg twice daily) • from11,0 kg to 15,5 kg: **27 mg twice daily** • from 15,6 kg to 20,0 kg: **36 mg twice daily** • from 20,1 kg to 24,5 kg: **45 mg twice daily** • from 24,6 kg to 29,0 kg: **54 mg twice daily** • from 29,1 kg to 33,5 kg: **63 mg twice daily** • from 33,6 kg to 38,0 kg: **72 mg twice daily** • from38,1 kg to 42,5 kg: **81 mg twice daily** • ≥ 42,6 kg: **90 mg twice daily**  age 16 or older :• **90 mg twice daily under skin** |
|  |  |  |  |  | age | | | kg | | | 2 | 27/05/2003 | adults |  |
|  |  |  |  |  | ≥ 6years | | | ≥ 11 | | |  | 27/05/2003 | >6 years |  |
| **Maraviroc (MVC)** | ≥ 2 years (EMA) | • **≥ 10 kg** | • from 10 kg to <20 kg: 50 mg  • from ≥20 kg to <30 kg: 75 mg | 2 | Oral solution (20mg/ml) | | | Tablet  150mg 300mg | | | n^o^ | date | age | Children:  • from 10 kg to <20 kg: 50 mgtwice a day with potent inhibitor CYP3A • from ≥20 kg to <30 kg: 75 mg twice daily with potent inhibitor CYP3A • from ≥30 kg to <40 kg: 100 mg twice daily with potent inhibitor CYP3A; 300 mg BID with other drugs  • ≥40 kg: 150 mg twice daily with potent inhibitor CYP3A; 300 mg BID with other drugs   Adults: • **150 mg twice daily (with potent inhibitor CYP3A)** • **300 mg twice daily (with other drugs )** • **600 mg twice daily (with potent inhibitor CYP3A)** |
|  |  |  |  |  | age | kg | | age | | kg | 2 | 18/09/2007 | >16 years |  |
|  |  |  |  |  | ≥ 2years | 10 | | ≥ 2years | | 10 |  | 30/10/2015 | 2 years |  |
| **Dolutegravir (DTG)** | ≥6 years (EMA) | • **≥ 15 kg** | • from 15 kg to <20 kg: 20 mg (2 tb X 10 mg) • from ≥20 kg to <30 kg: 25 mg | 1 | Tablet  10mg 25mg 50mg | | | | | | n^o^ | date | age | children between 6 and 12 years old: • from 15 kg to <20 kg: **20 mg once daily (2 cp da 10 mg)** • from ≥20 kg to <30 kg: **25 mg QD**  • from ≥30 kg to <40 kg: **35 mg once daily (1 cp da 25 mg + 1 cp da 10 mg)**  Adolescents, aged 12 to under 18 years with a body weight of at least 40 kg: • **1 tb of** **50 mg once daily, without resistance to the class of integrase inhibitors**   Adults, over 18 years of age:• **1 tb of 50 mg once daily** |
|  |  |  |  |  | age | | | kg | | | 2 | 16/01/2014 | >6 years |  |
|  |  |  |  |  | ≥ 6years | | | ≥ 15 | | |  |  |  |  |
| **Raltegravir (RAL)** | 4 week ≥ 2 yeras and ≤ 12 years(EMA) ≥ 6 years(EMA) | • **Granules for Oral Suspension:≥ 3 kg e ≤ 20 kg•Chewable Tablets ≥ 11 kg • Film-coated tablets > 25 kg** | Birth : 1,5 mg/kg/dose from 1°week to 4° week: 3 mg/kg/dose. ≥3kg to 25kg 6 mg/kg approximately>25 kg <28 150mg28 kg< 40kg 200≥40 kg: 300 mg | 222222 | Granules for Oral Suspension:(20 mg/ml) | | Chewable Tablets:(25 mg100 mg) | | Film-coated tablets:(400 mg600 mg) | | n^o^ | date | age | Granules for Oral Suspension **6 mg/kg/dose twice daily**, or:• 3 kg: **1 ml (20 mg) twice daily**• 4 kg - 5 kg: **1,5 ml (30 mg) twice daily**• 6 kg - 7 kg: **2 ml (40 mg) twice daily**• 8 kg - 10 kg: **3 ml (60 mg) twice daily**• 11 kg - 13 kg: **4 ml (80 mg) twice daily**• 14 kg - 20 kg: **5 ml (100 mg) twice daily**Chewable Tablets• 11 kg - 14 kg: **75 mg (3 tb of 25 mg) twice daily**• 14 kg - 19 kg: **100 mg (1 tb of 100 mg) twice daily**• 20 kg - 27 kg: **150 mg (1,5 tb of 100 mg) twice daily**• 28 kg - 40 kg: **200 mg (2 tb of 100 mg) twice daily**• ≥ 40 kg: **300 mg (3 tb of 100 mg) twice daily**Film-coated tablets:• **1 tb of 400 mg twice daily• 1 tb of 600 mg twice daily for naive patients with a weight ≥ 40 kg** |
|  |  |  |  |  | age | kg | age | kg | age | kg | 3 | 21/12/2007 | adult |  |
|  |  |  |  |  | birth | 3 - 20 | 2- 12 anni | ≥ 11 | ≥ 6 years | > 25 |  | 25/02/2013 15/06/2016 23/02/2018 | >2 years 4 weeks birth |  |
| **EVG/COBi + FTC + TDF** | > 12 years (EMA) | **>35kg** | 50 mg (EVG), 150 mg (COBi), 200 mg (FTC), 300 mg (TDF) | 1 | Tablet  150 mg (EVG), 150 mg (COBi), 200 mg (FTC), 300 mg (TDF) | | | | | | n^o^ | date | age | • **1 tb once daily** |
|  |  |  |  |  | age | | | kg | | | 1 | 24/05/2013 | 12 years |  |
|  |  |  |  |  | ≥ 18 years | | | --- | | |  |  |  |  |
| **DTG + ABC + 3TC** | > 12 years (EMA) | • > **40 kg** | : 50 mg (DTG), 600 mg (ABC), 300 mg (3TC) | 1 | Tablet   50 mg (DTG), 600 mg (ABC), 300 mg (3TC) | | | | | | n^o^ | date | age | • **1 tb once daily** |
|  |  |  |  |  | age | | | kg | | | 1 | 02/09/2014 | >12 years |  |
|  |  |  |  |  | ≥ 12yers | | | 40 | | |  |  |  |  |
| **EVG/COBi + FTC + TAF** | ≥ 12 ayears (EMA) | • > **35 kg** | 150 mg (EVG), 150 mg (COBi), 10 mg (TAF) 200mg (FTC) | 1 | Tablet   150 mg (EVG), 150 mg (COBi), 10 mg (TAF) 200mg(FTC) | | | | | | n^o^ | date | age | • **1 tb once daily** |
|  |  |  |  |  | age | | | kg | | | 1 | 19/11/2015 | >12 years |  |
|  |  |  |  |  | ≥ 12years | | | 40 | | |  |  |  |  |

**Table S2.** **Characteristics of children receiving lopinavir/ritonavir off-label**

| Patient | Age at the beginning of cART | Formulation | Daily Administrations  (n) | Current Cart | **Pre cART CD4^+^ Lymphocyte** Count cell/µL | **CD4^+^ Lymphocityes Count at last check** cell/µL | Pre-cART VL**^*^**  copies/mL | VL^*^ at last check copies/mL |
| --- | --- | --- | --- | --- | --- | --- | --- | --- |
| n°1 | **1** | Tablet | 2 | ABC+3TC+LPV+RTV | 567 | 1393 | N/A | U |
| n°2 | **0** | Missing | Missing | ABC+3TC+LPV+RTV | 664 | 1852 | 1000000 | 58 |
| n°3 | **1** | Missing | Missing | AZT+3TC+LPV+RTV | 672 | 1 | 35093 | U |
| n°4 | **1** | Oral syrup | 2 | AZT+3TC+LPV+RTV | 339 | 730 | 8970000 | U |
| n°5 | **1** | Oral syrup | 2 | ABC+3TC+LPV+RTV | 1116 | 1702 | 1000000 | U |
| n°6 | **2** | Oral syrup | 2 | ABC+3TC+LPV+RTV | 246 | 882 | 5400352 | U |
| n°7 | **1** | Oral syrup | 2 | ABC+3TC+LPV+RTV | 990 | 589 | 34691 | U |
| n°8 | **1** | Oral syrup | 2 | ABC+3TC+LPV+RTV | >=25%^*^ | 1957 | N/A | U |
| n°9 | **1** | Tablet | 2 | AZT+3TC+LPV+RTV | >=25%^*^ | 1656 | 144800 | U |
| n°10 | **0** | Oral syrup | 2 | AZT+3TC+LPV+RTV | >=25%^+^ | 1306 | N/A | U |
| ABC: abacavir; 3TC: lamivudine; LPV: lopinavir RTV: ritonavir; AZT: zidovudine. N/A: not available U: undetectable; D: detectable. *: we measured the value as a percentage, when the absolute value was not available. | | | | | | | | |

**Table S3. Characteristics of children receiving emtricitabine/ tenofovir disoproxil off-label (no child received on-label treatment in this group)**

| Patient | Age at the beginning of cART | Formulation | Daily  Administrations  (n). | Current cART | Pre cART CD4^+^ Lymphocytes Count cell/µL | CD4^+^ Lymphocityes Count at last check cell/µL | Pre-cART VL**^*^**  copies/mL | VL^*^ at last check copies/mL |
| --- | --- | --- | --- | --- | --- | --- | --- | --- |
| n°1 | **14 years*** | Tablet | 1 | **FTC+TDF**+DTG | 800 | 704 | U | U |
| n°2 | **14 years*** | Tablet | 1 | **FTC+TDF**+DTG | 15-25%^a^ | 184 | 26069 | 49714 |
| n°3 | **7 years** | Tablet | 1 | **FTC+TDF**+DRV+RTV | 1061 | 1355 | 1365 | U |
| n°4 | **12 years*** | Tablet | 1 | **FTC+TDF**+DRV+RTV | 720 | 662 | U | U |
| n°5 | **14 years*** | Tablet | 1 | **FTC+TDF**+MK0 | 1263 | 872 | 3576 | U |
| n°6 | **11 years** | Tablet | 1 | **FTC+TDF**+LPV+RTV | 15-24%^a^ | 346 | D | 83182 |
| n°7 | **13 years*** | Tablet | 1 | **FTC+TDF**+RTV+ATV | 395 | 483 | 107 | U |
| n°8 | **11 years** | Tablet | Missing | **FTC+TDF**+NVP | 670 | 908 | U | U |
| n°9 | **12 years*** | Tablet | 1 | **FTC+TDF**+NVP | 578 | 397 | U | U |
| n°10 | **14 years*** | Tablet | 1 | FTC+NVP+TDF | 521 | 592 | U | U |
| n°11 | **11 years** | Tablet | 1 | FTC+TDF+LPV+RTV | 559 | 800 | 5300 | U |
| Note. VL: viral load; FTC: emtricitabine; TDF: tenofovir disoproxil; DTG: dolutegravir; DRV: darunavir; RTV: ritonavir; NVP: nevirapine; LPV: lopinavir. U: undetectable; D: detectable. *Patients over 14 years old were considered off-label because when the therapy started the drug had been approved for an age over 18 years old or older. | | | | | | | | |

**Table S4. Characteristics of children receiving elvitegravir/cobicistat/emtricitabine/tenofovir alafenamide off-label**

| Patient | Age at the beginning of cART | Formulation | Daily  Administrions  (n) | Current cART | Pre cART CD4^+^ Lymphocyte Count cell/µL | CD4^+^ Lymphocityes Count at last check  cell/µL | Pre-cART VL**^*^**  copies/mL | VL^*^ at last check copies/mL |
| --- | --- | --- | --- | --- | --- | --- | --- | --- |
| n°1 | **11 years** | Tablet | 1 | FTC+TAF+EVG+COB | 960 | 710 | D | U |
| n°2 | **11 years** | Tablet | 1 | FTC+TAF+EVG+COB | 935 | 865 | U | U |
| n°3 | **8 years** | Tablet | 1 | FTC+TAF+EVG+COB | 822 | 1689 | 20000 | U |
| n°4 | **9 years** | Tablet | 1 | FTC+TAF+EVG+COB | 1068 | 892 | U | U |
| n°5 | **8 years** | Tablet | 1 | FTC+TAF+EVG+COB | 763 | 826 | U | U |
| FTC: emtricitabine; TAF: tenofovir alafenamide; EVG: elvitegravir; COB: cobicistat. VL: viral load; U: undetectable D: detectable | | | | | | | | |

**Table S5. Characteristics of children receiving abacavir/dolutegravir/lamivudine off-label**

| Patient | Age at the beginning of cART | Formulation | Daily  Administations  (n) | Current cART | Pre cART CD4^+^ Lymphocyte Count. cell/µL | CD4^+^ Lymphocityes Count at last check  cell/µL | Pre-cART VL**^*^**  copies/mL | VL^*^ at last check copies/mL |
| --- | --- | --- | --- | --- | --- | --- | --- | --- |
| n° 1 | **11 years** | Tablet | 1 | ABC+3TC+DTG | >=25%^*^ | 855 | U | U |
| n° 2 | **12 years** | Tablet | 1 | ABC+3TC+DTG | 15-24%^*^ | 553 | U | U |
| n° 3 | **12 years** | Tablet | 1 | ABC+3TC+DTG | 943 | 801 | 19 | U |
| n° 4 | **10 years** | Tablet | 1 | ABC+3TC+DTG | 1446 | 1791 | U | U |
| n° 5 | **12 years** | Tablet | Missing | ABC+3TC+DTG | >=25%^*^ | 753 | D | U |
| n°6 | **8 years** | Tablet | Missing | ABC+3TC+DTG | >=25%^*^ | 698 | U | U |
| n°7 | **11 years** | Tablet | Missing | ABC+3TC+DTG | 863 | 976 | 20 | 20 |
| n°8 | **10 years** | Tablet | Missing | ABC+3TC+DTG | 623 | 706 | U | U |
| ABC: abacavir; 3TC: lamivudine; DTG: dolutegravir.VL: viral load *; U: undetectable; D: detectable | | | | | | | | |
